# Investigating The Relationship Between Hippocampus:Dentate Gyrus Volume and Hypothalamus Metabolism in Participants with Major Depressive Disorder

**DOI:** 10.21203/rs.3.rs-2729363/v1

**Published:** 2023-04-03

**Authors:** Karen Lin, Daniel Sunko, Junying Wang, Jie Yang, Ramin Parsey, Christine DeLorenzo

**Affiliations:** Cornell University; Stony Brook University

## Abstract

Reduced hippocampal volume occurs in major depressive disorder (MDD), theoretically due to elevated glucocorticoids from an overactivated hypothalamus-pituitary-adrenal (HPA) axis. To examine this in humans, hippocampal volume, and hypothalamus (HPA axis) metabolism was quantified in participants with MDD before and after antidepressant treatment. 65 participants (n = 24 males, n = 41 females) with MDD were treated in a double-blind, randomized clinical trial of escitalopram. Participants received simultaneous positron emission tomography (PET) / magnetic resonance imaging (MRI) before and after treatment. Linear mixed models examined the relationship between hippocampus/dentate gyrus volume and hypothalamus metabolism. Chi-squared tests and multivariable logistic regression examined the association between hippocampus/dentate gyrus volume change direction and hypothalamus activity change direction with treatment. Multiple linear regression compared these changes between remitter and non-remitter groups. Covariates included age, sex, and treatment type. No significant linear association was found between hippocampus/dentate gyrus volume and hypothalamus metabolism. 62% (38 of 61) of participants experienced a decrease in hypothalamus metabolism, 43% (27 of 63) of participants demonstrated an increase in hippocampus size (51% [32 of 63] for the dentate gyrus) following treatment. No significant association was found between change in hypothalamus activity and change in hippocampus/dentate gyrus volume, and this association did not vary by sex, medication, or remission status. As this multimodal study, in a cohort of participants on standardized treatment, did not find an association between hypothalamus metabolism and hippocampal volume, it supports a more complex pathway between hippocampus neurogenesis and treatment response.

## Introduction

Major Depressive Disorder accounts for 10.3% of all disease burden worldwide.^1^ MDD’s prevalence is partially driven by a lack of knowledge regarding its pathophysiology, hindering treatment development. As stressful life events often precede depression, examining the brain’s stress response, specifically the hypothalamus in the hypothalamic-pituitary-adrenal (HPA) axis, could shed light on the biology of MDD and antidepressant treatment response.^2^ The hippocampus, while not explicitly part of the HPA axis, facilitates HPA-related responses, and is affected by stress.^2^

Within minutes of a stressor, the HPA axis initiates the autonomic stress response by stimulating parvocellular neurons in the paraventricular nucleus of the hypothalamus. Corticotropin-releasing factor is released, which in turn induces the release of adrenocorticotropic hormone, responsible for glucocorticoid synthesis in the adrenal cortex.^3^ Glucocorticoids, such as cortisol, range in function but play an important role in maintaining homeostasis after the stress stimulus. Specifically, to initiate the feedback loop that terminates the response, cortisol binds to glucocorticoid receptors (GR).^4,5^ The function of GRs, which provide negative feedback during stress^4^, can be disrupted by severe and prolonged stress. Prolonged exposure to cortisol also overworks immune responses and leads to the overproduction of proinflammatory cytokines. These proinflammatory cytokines influence GR function, typically by increasing expression of GRβ isoforms, which are inactive forms of GRs that compete with the active form GRa, causing glucocorticoid resistance.^6^ The sensitivity of GRs is also regulated by the chaperone protein FKBP5. Transcription of FKBP5 increases with circulating corticosteroids through a hormone response element within a promoter of the gene.^7,8^ FKBP5 binds to the GR complex, reducing its affinity for glucocorticoid binding, and decreasing the efficiency of GR translocation into the nucleus.^9^ The overall effect of these events is to hamper negative feedback, resulting in an overactive HPA axis.

The hippocampus, within the limbic system, plays an important role in memory^10^ and decision making.^11^ Reduced hippocampal volume is often associated with MDD^12^ and the glucocorticoid hypothesis suggests this may be due to a dysregulated HPA axis.^13^ Extended exposure to glucocorticoids causes dendritic retraction in the hippocampus, a reversible form of volume loss that causes the hippocampus to be vulnerable to cell death.^14^ Further, an animal model of an anxiety/depressive-like state suggests that cortisol hampers proliferation of progenitor cells in the hippocampus.^15^ Partially supporting this glucocorticoid hypothesis, patients with depression report a higher rate of stressful life events^16^ and long-term stress is associated with volume loss in the hippocampus.^17^A model used to study the glucocorticoid hypothesis is Cushing’s syndrome, where glucocorticoids are commonly overproduced, which is often coupled with a reduced hippocampal volume.^18^ This may be a particularly relevant model because MDD accompanies Cushing’s syndrome in 51–81% of cases.^19^

One study showed that reducing cortisol levels in Cushing’s syndrome resulted in normalization of hippocampus volume, suggesting a reversible volume decrease.^20^ Relatedly, a neurogenic hypothesis posits that neurogenesis is needed for recovery for MDD.^21^ Correspondingly, selective serotonin reuptake inhibitors (SSRIs), the most commonly prescribed antidepressant therapy^22^, have been shown to normalize HPA activity, resulting in lower glucocorticoid levels in rats^23^ and increased posterior hippocampal volume in human participants.^24^ Rodent models and retrospective analyses using postmortem tissue suggest this neurogenesis occurs in the dentate gyrus of the hippocampus.^25^

This study will examine the relationship between change in HPA activity and hippocampal volume with antidepressant treatment *in vivo*. It is a secondary analysis of a cohort with MDD designed to predict antidepressant treat response from imaging.^26^ In order to assess HPA activity frequently, cortisol levels are generally measured;^27^ however, these results can be confounded by cortisol level variation with the circadian cycle^28^ and cortisol was not acquired as part of the original study. 2-[^18^F]-fluorodeoxyglucose Positron Emission Tomography (FDG PET) imaging can be used to quantitatively assess glucose metabolism, which is a proxy for neuronal activity and remains relatively consistent throughout the day while the person is awake.^29,30^ We use FDG-PET to assess glucose uptake in the hypothalamus, a suggested proxy for HPA axis.^31^ Although, to our knowledge, FDG-PET has not been used in humans to probe HPA axis, FDG PET has been used in a rhesus monkey model to demonstrate that stress induces activation of the HPA axis.^32^

This study will compare hippocampal volume, including volume of the dentate gyrus specifically (as measured by MRI) to HPA axis metabolism (as measured by FDG-PET uptake in the hypothalamus) in participants with MDD, both before and after treatment (placebo-controlled, randomized, double-blind treatment trial of escitalopram). It is hypothesized that there will be an inverse relationship between HPA axis metabolism and hippocampal/dentate gyrus volume. In addition, it is hypothesized that a decrease in HPA axis metabolism, assessed in the hypothalamus, is required for hippocampal/dentate gyrus volume increase with treatment and is more likely to be observed in those who remit following treatment (regardless of treatment) than those who did not.

In addition, this study will examine whether these relationships differ between males and females. Females more frequently exhibit an overactive HPA axis and hypercortisolism than males.^33^ This may be one of the reasons that depression incidence is about twice as high in women compared to men.^34^ In female rats, a less robust negative feedback in the HPA axis and more dysregulation was associated with lower densities of mineralocorticoid receptors (MRs) and GRs and less glucocorticoid binding in the hypothalamus.^35^ MRs have relatively higher affinity to cortisol, are less specific in their binding and are able to regulate even low cortisol concentration thus mediating basal HPA axis activity. Lower densities of MRs and GRs hamper negative feedback and cortisol concentrations remain high, prolonging the stress response.^4^ Differences in estrogen, progesterone, and testosterone levels also affect glucocorticoid feedback.^34^ The concentration of GR mRNA has been shown to decrease with estrogen administration. The reduction in GRa may reduce the sensitivity of the female pituitary to negative feedback.^35^ Potentially for these reason, in a large sample of young adults, cortisol response indicated a healthy response to stress - a more rapid ascent and decline and a higher peak compared to females. In females, the slower resistance may lead to habituation after repeated exposures to stress.^36^ For these reasons, we hypothesize HPA (hypothalamus) metabolism will be higher in females and the relative volume of the hippocampus will also be smaller in females.

## Methods

This study, which was approved by the Institutional Review Board of Stony Brook University, included 85 participants who received pretreatment imaging (see CONSORT diagram^26^). Of these 85 participants, 65 participants (n = 24 males, n = 41 females, demographic and clinical information is provided in Table 1) had at least one useable PET scan. Reason for excluding PET imaging included participant withdrawal from the study, greater than 20% change in glucose during the imaging, diabetes, or excessive motion^26^. Of those 65 participants, n=1 pretreatment hippocampal/dentate gyrus volume was not used due to motion in the MRI and n=1 did not receive posttreatment imaging. Inclusion criteria consisted of: age of at least 18 years old, ability to provide signed informed consent. All participants were verified by a trained rater as having been diagnosed with MDD assessed via Structured Clinical Interview for DSM-IV (SCID-IV), and a score of 22 or higher on the Montgomery-Åsberg Depression Rating Scale^37^ (MADRS).TheMADRS was used only for inclusion of participants and was not used as an outcome measurement in this study to prevent inflation of symptoms and artificial treatment response. Potential participants were excluded in the event that they had a significant active physical illness, were currently undergoing successful antidepressant treatment, had significant neurological deficits, had a high potential for excessive substance use during the study period, had electroconvulsive therapy within six months, had current psychosis, had a lifetime history of bipolar disorder, had medical contraindications to escitalopram (study drug), such as failed escitalopram therapy of appropriate dose and duration in the past, or had contraindications to MRI or PET imaging, such as pregnancy or breast feeding. All participants were medication-free for three weeks prior to the study, either having completed medication washout (for ineffective medication) or by enrolling as psychotropic medication free. PET/MRI data from this cohort has been published in previous studies.^26,38,39^ None have examined the relationship between PET and MRI measures examined in this work.

Prior to, and following approximately 8 weeks of treatment, simultaneous PET/MRI imaging was acquired on a Siemens Biograph mMR. Through a double-blind design, participants were randomized to treatment with either placebo or escitalopram. Participants received 10 mg of escitalopram or placebo in week 1, 20 mg in week 2 and 3, and 30 mg in week 4–8. Placebo treatment followed the same design (one placebo pill was equivalent to one 10 mg escitalopram pill). This schedule was altered in cases of treatment intolerance (in both treatment cohorts). The Hamilton Depression Rating Scale 17 (HDRS-17) was administered in proximity to imaging. Remission was defined *a priori* as post-treatment HDRS-17 less than or equal to 7.^26^

### Magnetic Resonance Imaging (MRI)

A magnetization-prepared rapid gradient-echo (MP-RAGE) T1-weighted structural image was acquired with the following parameters: TR=2300ms, TE=3.24ms, flip angle=9 degrees, IPAT GRAPPA factor 2, FOV=223×210×195mm, bandwidth=220 Hz/Px, echo spacing=7.8ms, voxel size=0.87×0.87×0.87mm. T1 structural images were processed through automated hippocampal subfield segmentation pipeline of Freesurfer 5.3.0 (http://surfer.nmr.mgh.harvard.edu) to automatically extract the dentate gyrus as well as the whole hippocampus from the Desikan-Killiany atlas.^40,41^ The hypothalamus was delineatedvia nonlinear registration of the participant’s T1 image to the MNI template for the CTI168 high-resolution subcortical brain nucleus atlas^42,43^. Nonlinear warp parameters were generated using Advanced Normalization Tools (ANTs).^42,43^ For quality control, an overlay of the warped region on each participant’s MRI was visually inspected.

### Positron Emission tomography (PET)

PET images were collected for 60 min. Raw listmode PET data were reconstructed offline using Siemens’ e7 Tools software and a CT-like Boson MR-based attenuation map.^44,45^ Sinogram files were generated using the following frame definitions: 8×15sec, 6×30sec, 5×60sec, 4×300sec, and 3×600sec. Frames were corrected for motion^46^ and co-registered to MRI. Regions delineated on the MRI (see above) were transferred to the PET images through the co-registration. The Patlak graphical approach was used to estimate metabolic rate of glucose uptake (MRGlu) from the time activity curve while correcting for blood glucose and the lumped constant, using Simultaneous Estimation and a single venous sample, as previously described.^26^

### Statistical Analysis

Escitalopram and placebo subgroups were not examined separately as treatment response was not expected to differ.^47^ A previous study showed similar neurobiological changes in patients who achieved remission using either placebo or SSRI.^48^ However treatment status was still considered a covariate in all analyses.

Linear mixed models were utilized to examine the relationships between hippocampus/dentate gyrus volume and hypothalamus metabolism considering data from both pre- and post-treatment with age, sex, and pre/post-treatment timepoint as covariates. Two-way interactions between sex and hypothalamus metabolism as well as between pre- and post-treatment timepoint and hypothalamus metabolism were further examined to model the sex-specific relationships and pre- and post-specific relationships in separate models. For the hippocampus, Unstructured variance-covariance structures were selected with a smaller Akaike Information Criteria (AIC) than Compound Symmetric. For the dentate gyrus, Compound Symmetric variance-covariance structures were selected with a smaller AIC than Unstructured.

A chi-squared test with exact p-values based on Monte Carlo simulation was used to examine the marginal association between the categorical variable (sex) and treatment cohorts (medication type: escitalopram, placebo) as well as between hippocampal/dentate gyrus volume change direction (increase or decrease) and hypothalamus metabolism change direction (increase or decrease). Wilcoxon rank sum tests were used to compare unadjusted marginal differences for any continuous covariates (age, hypothalamus metabolism before and after treatment and percent change in hypothalamus metabolism) as well as continuous outcome variables (hippocampus/dentate gyrus volume before and after treatment and percent change in hippocampus/dentate gyrus volume) among two groups (escitalopram, placebo). Spearman rank correlation coefficient was used to measure the linear relationships between the percent change in hippocampus/dentate gyrus volume and percent change in hypothalamus metabolism by medication type. A multivariable logistic regression model was performed to model the association between hypothalamus metabolism change direction and hippocampus/dentate gyrus volume change direction adjusting for age, sex, and medication type. A twoway interaction between sex and hypothalamus metabolism change was further examined to model the sex-specific relationship.

A multiple linear regression model with percent change in hypothalamus activity interacted with remission status was implemented to compare the differences in the relationships between percent change in hypothalamus activity and hippocampus/dentate volume between remitter and non-remitter groups.

Statistical analysis was performed using SAS 9.4 and significance level was set at 0.05 (SAS Institute Inc., Cary, NC).

## Results

As displayed in Table 1, none of the examined outcome measures (hippocampus volume, hypothalamus metabolism) or covariates (age, sex) were statistically significantly different between the active medication and placebo groups. For this reason, they are combined in subsequent analyses (though treatment type remains as a covariate).

### Relationship between hypothalamus metabolism and hippocampal volume

There was no strong evidence for a significant linear relationship between hippocampus volume and hypothalamus metabolism (estimated coefficient = −56.5, 95% CI: [−231.0, 118.0], p-value = 0.52), adjusting for age, sex, and pre and post treatment timepoint. Female participants had significantly lower hippocampus volume than male participants (p-value < 0.01), adjusting for age, hypothalamus metabolism and pre and post treatment timepoint. Hippocampus volume had a significantly negative relationship with age (p-value < 0.01), adjusting for sex, hypothalamus metabolism and pre and post treatment timepoint. There was no significant difference between female and male hypothalamus metabolism (p-value = 0.11), while a significantly negative relationship with age was also found (p-value = 0.01).

Similarly, no strong evidence existed to show a significant linear relationship between dentate gyrus volume and hypothalamus metabolism (estimated coefficient = −8.7, 95% CI: [−40.9, 23.6], p-value=0.59), adjusting for age, sex and pre and post treatment timepoint. Female participants had significantly lower dentate gyrus volume than male patients (p-value < 0.01), adjusting for age, hypothalamus metabolism and pre and post treatment timepoint. However, dentate gyrus volume was not significantly associated with age (p-value = 0.14), adjusting for sex, hypothalamus metabolism and pre and post treatment timepoint.

Neither of the two interaction terms, examined in separate linear mixed models, were significant, which indicated the linear relationship between hippocampus volume (or dentate gyrus volume) and hypothalamus metabolism was not significantly different by sex or time point (pre-treatment or posttreatment).

### Relationship between change in hypothalamus activity and change in hippocampal volume with treatment

Table 2 shows no strong evidence for a relationship between a change in hypothalamus metabolism and a change in hippocampal (or dentate gyrus) volume based on the Chi-squared test, meaning a decrease in hypothalamus metabolism was not associated with an increase in hippocampal (or dentate gyrus) volume. A multiple linear regression model also showed no significant linear relationship between percent change in in hypothalamus metabolism and percent change in hippocampus/dentate gyrus volume (hippocampus: estimated coefficient = −0.02, 95% CI: [−0.08, 0.04], p-value = 0.51; dentate gyrus: estimated coefficient = −0.03, 95% CI: [−0.11, 0.06], p-value = 0.50) adjusting for age, sex and medication type.

However, as seen in Table 2, a slightly higher percentage of those who experienced a decrease in hypothalamus metabolism following treatment had an increase in hippocampal/dentate gyrus volume and a slightly higher percentage of those who experienced an increase in hypothalamus metabolism had a decrease in hippocampal volume. Based on this, an odds ratio was calculated based on different multivariable logistic regression models and it was found that participants with decreased hypothalamus metabolism were estimated to have a larger, but not statistically significant, chance of having an increase in hippocampus/dentate gyrus volume than participants with increased hypothalamus metabolism after adjusting for age, sex and medication type (hippocampus: OR=1.87>1, 95% CI: [0.6, 5.9], p-value = 0.28; dentate gyrus: OR = 1.12>1, 95% CI: [0.4, 3.6], p-value = 0.85).

The sex interaction term was further examined using a multivariable logistic regression model and was not significant, indicating that the association between hippocampus/dentate gyrus volume change and hypothalamus metabolism change was not significantly different across sexes, adjusting for age and medication type as covariates. Similarly, in the multiple linear regression model, the linear relationship between percent change in hypothalamus metabolism and percent change in hippocampus/dentate gyrus volume was not significantly different by sex, adjusting for age and medication type as covariates.

### Relationship between hippocampus volume and hypothalamus activity remitters versus non-remitters

The relationship between percent change in hypothalamus metabolism and percent change in hippocampus/dentate gyrus volume did not differ significantly by remission status (hippocampus: p-value=0.87, dentate gyrus: p-value = 0.42, Figure 2).

## Discussion

SSRIs have been shown to both normalize HPA activity, lowering glucocorticoid levels^23^ and increase hippocampal volume. Although examining both variables together prevents the determination of directionality, it was hypothesized that, with treatment, a decrease in hypothalamus metabolism (i.e., reduced HPA axis activity) would be associated with hippocampal volume increases. Therefore, in this study, we aimed to determine the relationship between HPA activity, as assessed through hypothalamus metabolism from PET, and hippocampal / dentate gyrus volume, assessed through MRI, acquired simultaneously. The dentate gyrus within the hippocampus was examined because SSRIs are thought to reverse the hippocampal volume atrophy by increasing neurogenesis specifically in the dentate gyrus.^24^ Examining dentate gyrus volume may therefore provide higher resolution than examining hippocampus volume as a whole. However, as can be seen in Figure 2, the range of percent differences are similar.

Correspondingly, we found no differences in outcome when examining the whole hippocampus versus the dentate gyrus separately.

Although half the participants in this cohort were randomized to placebo treatment, as previously published, remission rates did not differ across treatment type^26^, and as seen in this work, nor did change in hippocampal volume or hypothalamus metabolism, therefore groups were examined together, with treatment type as a covariate. This is consistent with a previous study showing similar metabolic changes in the brain with SSRI and placebo.^48^

**Figure 1:**
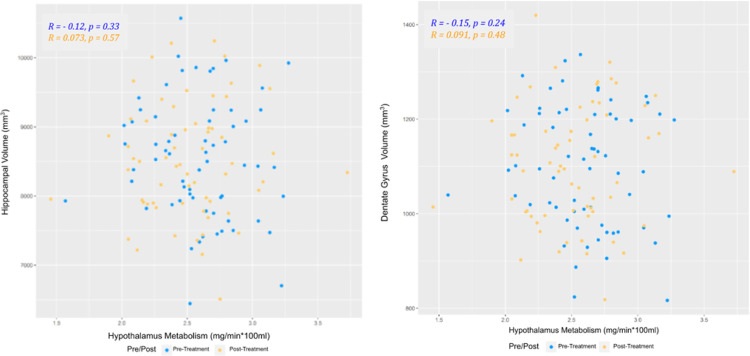
Scatterplots for hippocampus (Left) or dentate gyrus (Right) volume versus hypothalamus metabolism including pre-treatment (Blue) and post-treatment (Orange)

**Figure 2:**
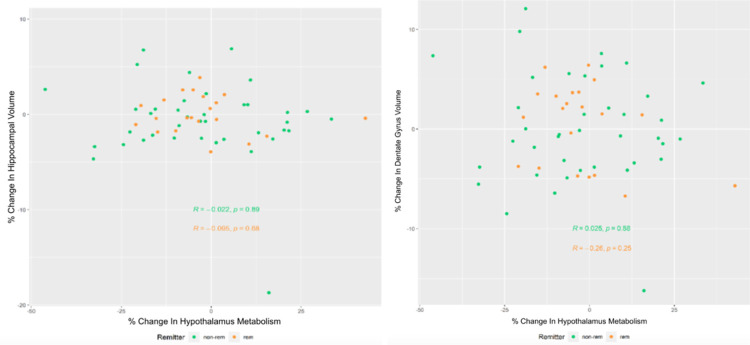
Scatterplot for percent change in hippocampus (Left) or dentate gyrus (Right) volume versus percent change in hypothalamus metabolism including non-remitters (Green) and remitters (Orange)

As the scatterplots in Figure 1 reveal, no association between hippocampus/dentate gyrus volume and hypothalamus metabolism was observed, before or after treatment or in the combined cohort. In Table 2, it was shown that, following treatment, although participants who experienced a decrease in hypothalamus metabolism had a higher chance of hippocampus/dentate gyrus volume increase than participants with an increase in hypothalamus metabolism, the result was not statistically significant. The cohort was then broken down into those who remitted and those who did not (Figure 2) in case the relationship was only apparent in those who recovered from the treatment. However, when separating remitters and non-remitters, still no significant relationship was found.

It is possible that the relationship exists as hypothesized but a larger sample size is required to detect it. Given that the current sample contains >60 participants, this suggests the effect size may be too weak to be clinically relevant. Rather, it is more likely that the consistent evidence above, examined in multiple ways using multiple modalities, suggests that the relationship between hypothalamus activity and hippocampus volume is complex and/or may not be linear. For example, it is possible that the hippocampal size is related to factors other than, or, in addition to HPA axis activity (see Limitations). This may be why the direction of our hypothesized association was confirmed (i.e., those who experienced a decrease in hypothalamus metabolism had a higher chance of hippocampus/dentate gyrus volume increase), but was not significant.

It is also interesting to note, on average, hypothalamus metabolism changes were higher than hippocampal volume changes. The range of change are: hippocampus (−18.7% and 9.8%), dentate gyrus (−16.2% and 12.1%), and hypothalamus metabolism (−46.1% and 42.9%). Although previous studies have reported volume changes over the course of weeks, it may be that metabolism changes occur more quickly and precede volume changes in this cohort, and that volume changes would occur over a longer time period This is consistent with previous analysis showing that most metabolic changes occur prior to those of structural.^49^ Yet, this is one motivation behind examining remitters and non-remitters separately. Any changes related to treatment response should be captured in this analysis, at least in those who remitted. Therefore, neurobiological changes that occur outside this treatment window may not be related to response.

### Limitations:

There are several limitations to this study: Previous studies have found that the mean age at onset of the first depressive episode was 37.4.^50^ As the average age of our sample is 29.8, it may not be a completely representative sample of the population of individuals with MDD. As neurobiology changes with age, the treatment-induced changes, and specifically the relationship between HPA axis activity and neurogenesis may change with age. However, as neurogenesis declines with age^51^, a younger age range may be the best population in which to examine this association. Another limitation involves the use of measurements of glucose uptake in the hypothalamus as a proxy for HPA axis activity. Since FDG-PET has not been used previously in humans to probe HPA axis, and because the limits of PET resolution prevent accurate quantification of activity in the pituitary and adrenal regions, it may not be the optimal method to assess HPA axis activity. The most used method of HPA axis sampling is measuring cortisol in blood.^31^ However, these measures may be influenced by ultradian and circadian rhythms.^31^ Additionally, the HPA axis is directly and indirectly controlled by multiple brain regions. The dorsomedial and ventromedial hypothalamus are direct controls, but so are the ventrolateral medulla and the nucleus tractus solitarius. Indirect controls include the medial prefrontal cortex, hippocampus, amygdala, and septum.^31^ Further, one study demonstrated that the stimulation of the glutamatergic path connecting the entorhinal cortex to the dentate gyrus facilitates the increase in long-term potentiation during adult hippocampus neurogenesis.^52^ Correspondingly, stimulation of entorhinal cortex-dentate gyrus circuitry has been shown to be antidepressive.^53^ Therefore, examining the hypothalamus in isolation may not provide enough information for assessing HPA activity. Future work investigating the upstream hippocampal circuitry, including the entorhinal cortex, and its involvement in the regulation of neurogenesis of the hippocampus as well as the association with hypothalamus metabolism can provide further insight into the relationship with hippocampal neurogenesis and the pathophysiology of MDD. Finally, based on the above evidence, those exposed to prolonged stress, and potentially those with MDD, experience dysfunction in the negative feedback mechanism that terminates the stress response. However, this improper termination may affect the length of the response more than the magnitude. Therefore, measuring the length of elevated hypothalamus activity may be more informative than measuring single in the magnitude. Glucocorticoids, being steroid hormones, remain in circulation longer when bound to transport proteins that extend their half-life.^54^

Despite the limitations, this study also had several strengths. A major strength of this study includes a rigorous statistical analysis of a large sample size of patients with MDD with PET and MRI data. To our knowledge, no study has investigated the relationship between the hypothalamus activity and hippocampus volume with treatment utilizing multimodal brain imaging techniques, PET, and MRI, allowing simultaneous assessment of both metabolism and structure, respectively. This is critically important to evaluate to further understand the mechanism of action of antidepressant treatment. Our investigation also includes covariates such as age and sex, with known effects on metabolism and volume, although we present no evidence to show that sex affects the relationship between hypothalamus metabolism and hippocampal volume. This is especially interesting given the known sex differences in these systems. Further, we provide insight into both hypothalamus metabolic and hippocampal volume changes with an active treatment (escitalopram) versus placebo. Consistent with previous evidence, we see no differences in the effects of these two treatments, even when accounting for remission status. Finally, results of our study provide a pathway for future research. Given the difficulty of replicating significant findings in biomedical research, the publication of negative results from well-powered studies is imperative for prevent overuse of resources in similar analyses.^55^ Specifically, as this thorough, well-powered investigation failed to find a relationship between hypothalamus metabolism and hippocampal volume, future work should instead include a more comprehensive approach (e.g., include regions in addition to the hypothalamus and potentially involvement of neurotransmitters).

## Conclusions

No statistically significant association was found between hypothalamus metabolism and hippocampus/dentate gyrus volume; therefore, the complexity of treatment-induced hippocampal volume changes likely requires examination of a more comprehensive circuitry of anatomy. Interestingly, however, no sex differences were found in these relationships suggesting that at least the relationship between hypothalamus activity and hippocampus volume does not underlie sex-effects in this system.

## Figures and Tables

**Table 1:** Descriptive table for each variable by medication type; Median+/−interquartile range are reported. Pre: before treatment; Post: after treatment

Variable	Total	Escitalopram	Placebo	P-value
Age (n = 65)	23.3±12.2	23.1±27.3	23.5±8.6	0.47
Sex: Female (n = 41)	41 (63.1%)	19 (46.3%)	22 (53.7%)	0.78
Hippocampus Volume Pre (mm^3^) (n = 64)	8514.2±1185.5	8414.1±868.9	8753.5±1314.8	0.25
Hippocampus Volume Post (mm^3^) (n = 64)	8461.5±1098.0	8318.4±1035.2	8554.1 ±1489.9	0.35
Hippocampus Volume Change (%) (n = 63)	−0.4±3.6	−0.4±3.5	−0.5±3.4	0.86
Dentate Gyrus Volume Pre (mm^3^) (n = 64)	1098.5±210.8	1091.9±188.8	1118.4±227.5	0.49
Dentate Gyrus Volume Post (mm^3^) (n = 64)	1097.8±192.3	1090.8±170.3	1107.7±234.3	0.33
Dentate Gyrus Volume Change (%) (n = 63)	0.0±7.5	−0.1±7.4	0.9±8.5	0.93
Hypothalamus Metabolism Pre (mg/min*100ml) (n = 64)	2.6±0.4	2.7±0.5	2.5±0.4	0.18
Hypothalamus Metabolism Post (mg/min*100ml) (n = 62)	2.5±0.5	2.5±0.5	2.6±0.5	0.28
Hypothalamus Metabolism Change (%) (n = 61)	−3.0±24.0	−6.1±20.4	−0.3±22.4	0.10

**Table 2:** Univariate analysis between hippocampus (Hip)/dentate gyrus (DG) volume change and hypothalamus metabolism change

			Hip volume change		
Variable	Change	Total	Decrease	Increase	P-value
hypothalamus metabolism change	Decrease	38 (62.3%)	21 (58.3%)	17 (68.0%)	0.59
	Increase	23 (37.7%)	15 (41.7%)	8 (32.0%)	
			DG volume change		
Variable	Change	Total	Decrease	Increase	P-value
hypothalamus metabolism change	Decrease	38 (62.3%)	18 (60.0%)	19 (63.3%)	1.00
	Increase	23 (37.7%)	12 (40.0%)	11 (36.7%)	
